# Endogenous CRISPR-assisted microhomology-mediated end joining enables rapid genome editing in *Zymomonas mobilis*

**DOI:** 10.1186/s13068-021-02056-z

**Published:** 2021-10-24

**Authors:** Xin Sui, Xiaojie Wang, Tao Liu, Qing Ye, Bo Wu, Guoquan Hu, Shihui Yang, Mingxiong He, Nan Peng

**Affiliations:** 1 State Key Laboratory of Agricultural Microbiology, Hubei Hongshan Laboratory, College of Life Science and Technology, Huazhong Agricultural University, Wuhan, 430070 Hubei People’s Republic of China; 2 Key Laboratory of Development and Application of Rural Renewable Energy (Ministry of Agriculture), Biomass Energy Technology Research Centre, Biogas Institute of Ministry of Agriculture, Chengdu, 610041 Sichuan People’s Republic of China; 3 State Key Laboratory of Biocatalysis and Enzyme Engineering, Hubei Engineering Research Center for Bio-Enzyme Catalysis, Environmental Microbial Technology Center of Hubei Province, Hubei Collaborative Innovation Center for Green Transformation of Bio-Resources, School of Life Sciences, Hubei University, Wuhan, 430062 People’s Republic of China

**Keywords:** *Zymomonas mobilis*, CRISPR-Cas, Microhomology-mediated end joining, Genome editing, DNA repair

## Abstract

**Background:**

*Zymomonas mobilis* is a natural ethanologen with many desirable characteristics, making it an ideal platform for future biorefineries. Recently, an endogenous CRISPR-based genome editing tool has been developed for this species. However, a simple and high-efficient genome editing method is still required.

**Results:**

We developed a novel gene deletion tool based on the endogenous subtype I–F CRISPR-Cas system and the microhomology-mediated end joining (MMEJ) pathway. This tool only requires a self-interference plasmid carrying the mini-CRISPR (Repeat–Spacer–Repeat) expression cassette, where the spacer matches the target DNA. Transformation of the self-interference plasmid leads to target DNA damage and subsequently triggers the endogenous MMEJ pathway to repair the damaged DNA, leaving deletions normally smaller than 500 bp. Importantly, the MMEJ repair efficiency was increased by introducing mutations at the second repeat of the mini-CRISPR cassette expressing the guide RNA. Several genes have been successfully deleted via this method, and the phenotype of a σ^28^ deletion mutant generated in this study was characterized. Moreover, large fragment deletions were obtained by transformation of the self-interference plasmids expressing two guide RNAs in tandem.

**Conclusions:**

Here, we report the establishment of an efficient gene deletion tool based on the endogenous subtype I–F CRISPR-Cas system and the MMEJ pathway in *Zymomonas mobilis*. We achieved single gene deletion and large-fragment knockout using this tool. In addition, we further promoted the editing efficiency by modifying the guide RNA expression cassette and selecting lower GC% target sites. Our study has provided an effective method for genetic manipulation in *Z. mobilis*.

**Supplementary Information:**

The online version contains supplementary material available at 10.1186/s13068-021-02056-z.

## Background

As the global environment continues to deteriorate and resources are gradually depleted, the development of alternative and eco-friendly resources that can produce biofuels is imminent. *Zymomonas mobilis* is a Gram-negative, facultative anaerobic ethanol-producing strain with many desirable characteristics, such as high specific productivity, high alcohol tolerance, lower biomass production, and a wide production pH range (pH 3.5–7.5) [1, 2]. Its unique Entner–Doudoroff (ED) pathway makes it an ideal strain for both metabolic engineering and commercial-scale production of bio-products, especially for the economic production of lignocellulosic biofuels and biochemical [3, 4].

Genome editing tools are the cornerstones for engineering the industrial microorganisms to achieve high production efficiency or to integrate novel pathways [5]. To develop *Z. mobilis* as a model microbe for synthetic biology and biorefinery applications, a series of genome-editing approaches have been explored. These approaches include classical chemical mutagenesis and adaptation, transposon mutagenesis, shuttle vectors and transformation approaches [6–9]. Recently, Clustered Regularly Interspaced Short Palindromic Repeats and its associated protein (CRISPR-Cas) systems were widely developed as genomic editing tools in bacteria [10, 11]. The CRISPR-Cas system is composed of CRISPR arrays and an endonuclease protein or several endonuclease proteins, whose DNA-targeting specificity and cutting activity can be programmed by a short guide RNA [12]. Currently, type II CRISPR-Cas9 system and type V Cpf1 system have been applied widely in eukaryotes and prokaryotes due to their advantages of a single effector protein [10, 13]. However, the intrinsic toxicity of Cas9 and Cpf1 might lead to cell death in some strains [14, 15]. While, the endogenous type I Cas systems are most frequent present in bacterial genomes [16]. Thus, the development of endogenous type I CRISPR-Cas system-based genome-editing tools might help to improve the efficiencies. Recently, heterologous CRISPR-Cas9 system [17], CRISPR-Cpf1 [18] and endogenous subtype I–F CRISPR–Cas system [19] have been used to edit *Z. mobilis* genome. The endogenous I–F system showed higher efficiency than the heterogenous CRISPR-Cas9 or Cpf1 systems in *Z. mobilis* [19], providing a powerful toolkit for diverse genome engineering purposes, including gene mutation, large-fragment deletion, and simultaneous multiple gene editing, which greatly benefit further study in this species*.*

In general, target DNA cleaved by CRISPR nucleases can be repaired via homology-directed repair (HDR), non-homologous end joining (NHEJ) pathway [20] or microhomology-mediated end joining (MMEJ) pathway [21–23]. The error-prone NHEJ repair system is often most prevalent in eukaryotes rather than prokaryotic genomes [24]. Therefore, HDR pathway is widely used for genome-editing in prokaryotes, but requiring cloning of additional homologous arms in editing plasmids, which hinders quick assessment of the gene functions in vivo. Recently, we have demonstrated that a MMEJ pathway present in *Z. mobilis* efficiently repaired the endogenous CRISPR-mediated genomic DNA damage [25]. In this study, we developed a genetic tool based on the endogenous CRISPR-Cas system and the MMEJ pathway to manipulate the genes in vivo by transformation of a shuttle vector only carrying the “Repeat-Spacer-Repeat” expression cassette, providing a simple and quick method to evaluate the gene functions in vivo.

## Results

### Endogenous CRISPR-Cas system and MMEJ pathway conferred high-efficient DNA deletions in *Z. mobilis*

**Fig. 1:**
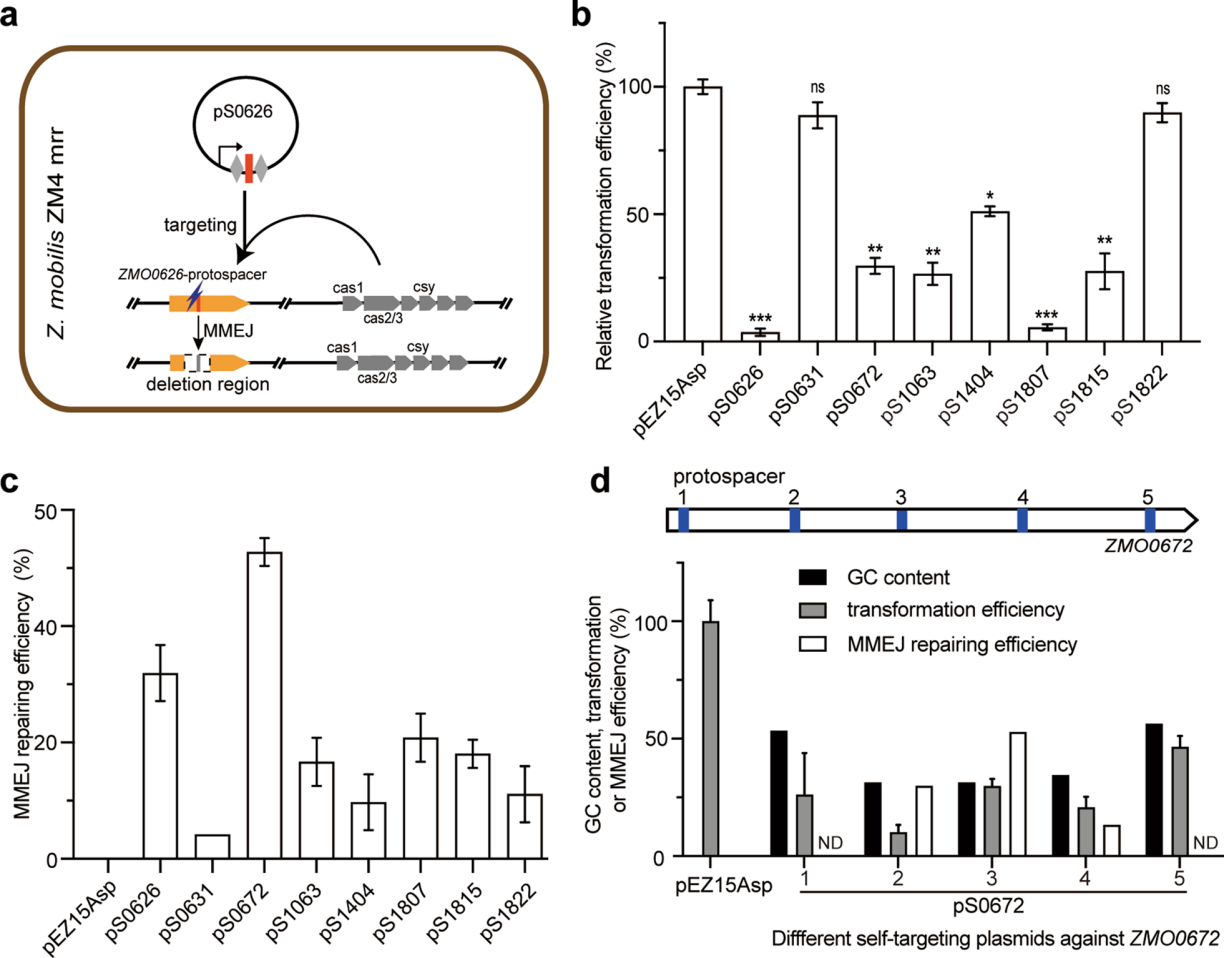
Establishment of a gene deletion method based on CRISPR-Cas system and MMEJ pathway in *Z. mobilis*. **a** Experimental paradigm for the gene deletion method. A self-targeting plasmid pS0626 (0626 relative to the target gene *ZMO0626*) carrying the expression cassette of “Repeat-Spacer-Repeat”, where the spacer matches the target DNA sequence followed a NCC PAM in *ZMO0626* as an example, is electroporated into *Z. mobilis* ZM4mrr cells. The guide RNA transcribed and processed from the “Repeat-Spacer-Repeat” cassette guides DNA cleavage at the target by the endogenous subtype I–F surveillance complex, and the cleaved DNA is subsequently repaired through MMEJ pathway. **b** Relative transformation efficiency of each self-interference plasmid against *ZMO0626*, *ZMO0631*, *ZMO0672*, *ZMO1063*, *ZMO1404*, *ZMO1807*, *ZMO1815* and *ZMO1822* genes, relative to the empty plasmid pEZ15Asp. Error bars represent the SD of three independent experiments. The significance was determined using a *t* test; *p* < 0.05 *, *p* < 0.01 **, *p* < 0.001 ***. **c** MMEJ efficiencies of the randomly selected colonies carrying each self-targeting plasmids. Error bars represent the SD of three independent experiments. **d** GC content of the protospacers affected the MMEJ repair efficiency after CRISPR interference. The locations of five protospacers on *ZMO0672* gene with different GC content were shown above, and GC content, transformation efficiency, MMEJ efficiency were shown below

Recently, we have found that self-targeting at the chromosome DNA via the endogenous subtype I–F CRISPR-Cas system led to high efficient MMEJ-mediated deletions covering the protospacers in *Z. mobilis* [25]. This raises a possibility to establish a gene deletion tool using the endogenous subtype I–F CRISPR-Cas system and MMEJ pathway (Fig. 1a). Here, eight genes encoded by *Z. mobilis* ZM4mrr were selected as the target genes to test this editing method. These genes include *ZMO0626* and *ZMO1404*, which encode the sigma factors σ^28^ and σ^70^, respectively. *ZMO0631* gene encodes ZraR protein, a transcriptional activator that acts on of σ^54^-RNA polymerase holoenzyme [26]. The *ZMO0672* gene encodes a DNA repair protein UvrC, and *ZMO1063* encodes a phage shock protein PspA. *ZMO1807* encodes a TonB-dependent receptor, and *ZMO1815* and *ZMO1822* encode two TonB-dependent siderophore receptors.

We constructed several self-targeting plasmids carrying the “Repeat-Spacer-Repeat” expression cassettes, where the “Spacer” sequences matched the protospacers at 8 target genes, respectively. For example, the pS0626 plasmid encodes the self-targeting spacer against the *ZMO0626* gene (Fig. 1a). The self-targeting plasmids were transformed into *Z. mobilis* ZM4mrr and showed significantly lower transformation efficiencies compared with the control empty vector pEZ15Asp, except the self-targeting plasmids pS0631 and pS1822 against *ZMO0631* and *ZMO1822*, respectively (Fig. 1b). 24 single colonies of each transformation were selected for colony PCR amplification of the target loci. The sequencing results showed that CRISPR interference at different target genes gave different MMEJ repair efficiencies in the single colonies of different transformants, ranging from 4.2 to 52.8% (Fig. 1c). We found no significant correlation between transformation efficiency and MMEJ efficiency, so we proposed that MMEJ efficiency varies at different locations of the same gene locus. Thus, five protospacers with different GC content on *ZMO0672* gene were selected as the targets (Fig. 1d). Transformation of the interference plasmids against these protospacers resulted in different transformation efficiencies (10.0–46.4%) compared with empty vector pEZ15Asp (Fig. 1d). It is particularly important that interference at the protospacers with higher GC content (e.g., protospacer 1 and 5: 51% and 53%, respectively) resulted in no MMEJ-mediated deletions (Fig. 1d). Similarly, we test more MMEJ-mediated deletions at different protospacers with different GC content in *ZMO0626*, *ZMO1807*, *ZMO1815*, *ZMO1822* genes, respectively. In addition, the correlation between GC content and MMEJ repair efficiency presented by them were consistent with that of *ZMO0672*: MMEJ efficiencies of corresponding targeted protospacers decrease with the increasing of GC contents (Additional file 4: Figure S4). These results infer that high GC content of the protospacers reduced the efficiency of MMEJ-mediated deletion in *Z. mobilis*.

### Engineered repeat sequence in the editing plasmid improved MMEJ efficiency

**Fig. 2:**
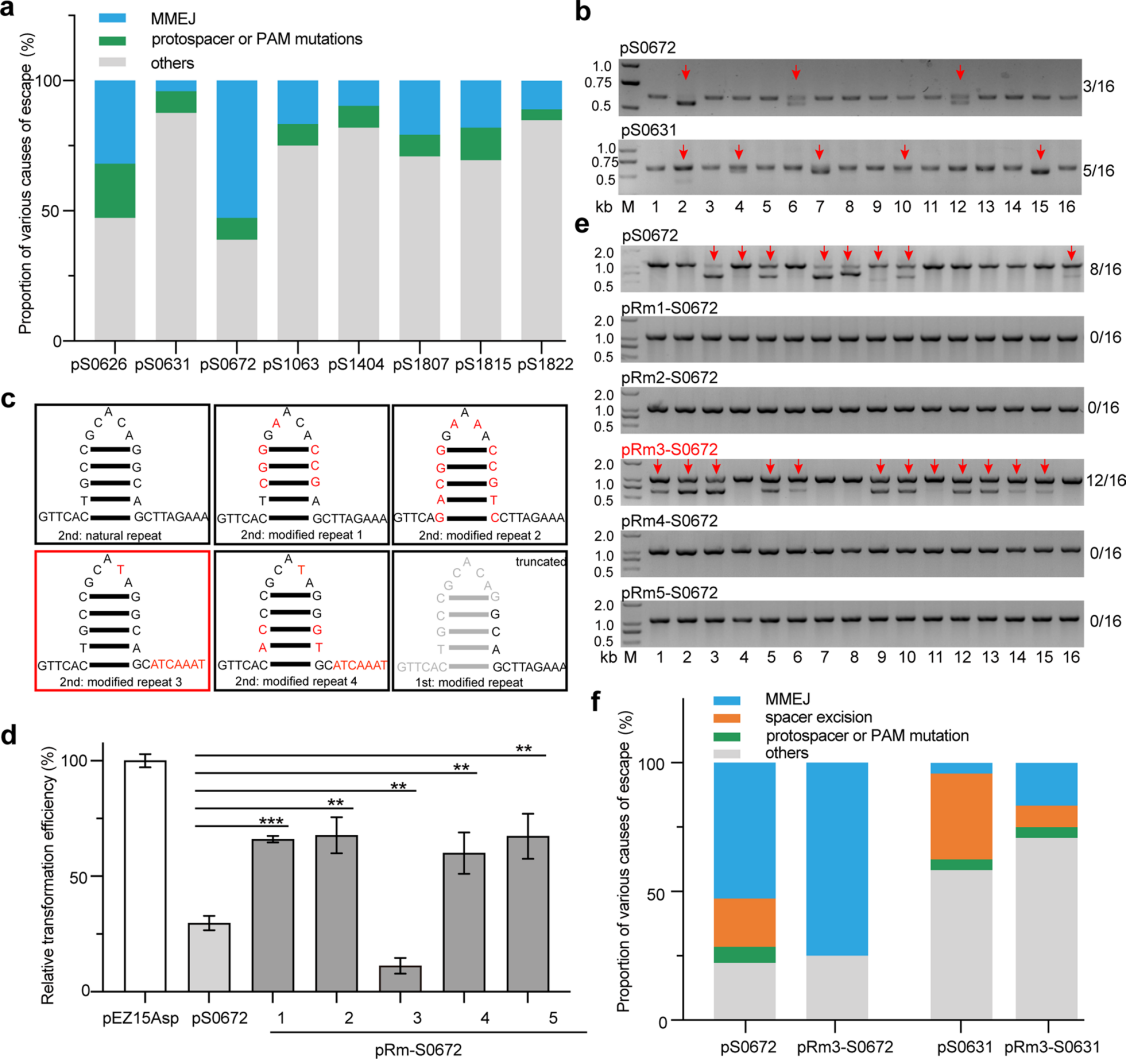
Optimization of CRISPR-Cas-assisted and MMEJ-directed genomic editing. **a** Portion of MMEJ deletion at the targets and mutations at the targets or editing plasmids in the transformants carrying the editing plasmids. MMEJ: the genomes showed MMEJ mediated deletions covering the protospacers; protospacer mutations: point mutations observed at the protospacer sequences; others: mutations at the mini-CRISPR (Repeat–Spacer–Repeat cassette) on the editing plasmids, or at other genes resulted in loss of CRISPR function, etc. **b** PCR amplification and agarose gel analysis spacer excision at the mini-CRISPR of pS0672 (above) and pS0631 (below) plasmids on 1.5% agarose gel. Bands, marked by red arrows, indicated excision of the spacer. **c** Sequences and predicted structures of the natural and modified repeats of the “Repeat-Spacer-Repeat” cassette. The mutation at the 1st or the 2nd repeat was indicated. Specifically, the modified nucleotides were shown in red, and the truncated nucleotides were shown in grey. **d** Transformation efficiencies of the control plasmid and different interference plasmids. The significance was determined using a *t* test; *p* < 0.01 **, *p* < 0.001 ***. **e** Colony PCR screening of MMEJ mutants transformed with the interference plasmids carrying different Repeat sequences against the *ZMO0672* gene. The lanes representing the transformants carrying MMEJ deletions were indicated by red arrows. **f** Portion of MMEJ deletion at the targets and mutations at the targets or editing plasmids in the transformants carrying the modified editing plasmids. Category “spacer excision” is included in the “others” in **a**

We have found that the MMEJ-mediated deletion efficiencies of the transformants ranged from 4.2 to 52.8% (Fig. 2a), which means half or more transformants carrying the editing plasmids have escaped CRISPR interference and the subsequent MMEJ repair. To understand how the transformants lacking MMEJ-directed deletions escaped self-targeting, we first analysed the DNA sequences of the target regions. We found that 8.3–20.8% transformants carried mutations at the PAM or protospacers (Fig. 2a), which is a common way for archaea and bacteria to escape CRISPR interference and, therefore, formation of colonies on the selection plates. Other transformants are presumed to carry mutations on the interference plasmids or at the *cas* genes to escape CRISPR interference (Fig. 2a). Then we extracted and tested the plasmids from the transformants carrying pS0672 and pS0631plasmids as examples. Agarose gel separation of the PCR products amplifying the mini-CRISPR showed smaller size bands corresponding to the deletions (Fig. 2b), and sequencing of the smaller bands indeed confirmed the deletion of “Spacer” at mini-CRISPR cassette. In the same time, the target gene locus of the transformants carrying the mutated plasmids also showed no MMEJ-mediated deletions by sequencing of the PCR products amplifying the *ZMO0672* and *ZMO0631* genes, suggesting escape of CRISPR interference through the deletion of “spacer”.

We proposed that the deletions of spacer probably occurred due to recombination at the two “Repeat” sequences in the mini-CRISPR cassette (Additional file 1: Figure S1a). Therefore, we introduced mutations into the first or the second “Repeat” sequences of the pS0672 plasmid, which either affected the stem structure or the loop structure (Fig. 2c), and tested the interference efficiency and the subsequent MMEJ efficiency after transformation of the mutated plasmids. Four engineered plasmids with large mutations at the stem structure of the second repeat (pRm1-S0672, pRm2-S0672 and pRm4-0672) and truncation of the first repeat (pRm5-0672) showed significant higher transformation efficiencies comparing to the original pS0672 (*p* < 0.05) and detected no MMEJ event (Fig. 2d, e), which means inhibition of CRISPR interference. However, the engineered plasmid pRm3-S0672 carrying mutations at the 3′ handle sequence of the second repeat (Fig. 2c) significantly promoted MMEJ efficiency via reducing recombination between the two “Repeat” sequences. 8 of 16 MMEJ-repaired transformants and 12 of 16 MMEJ-repaired transformants were detected after original plasmid pS0672 and modified plasmid pRm3-S0672 transformation (Fig. 2e). The modified plasmid also used for editing *ZMO0631*, the gene with the lowest MMEJ efficiency, named pRm3-S0631. Compared with pS0631, MMEJ efficiency of pRm3-S0631 was remarkable increased from 4.2 to 16.7% (Fig. 2f). Sequencing results of the mini-CRISPR cassettes in self-targeting plasmids of the transformants revealed that spacer excision from the mini-CRISPR cassettes were largely reduced in both pRm3-S0672 and pRm3-0631 plasmids, compared with their original plasmids, respectively (Additional file 1: Figure S1b). In brief, introducing one mutated nucleotide in the “loop” and seven mutated nucleotides in the 3’ handle sequences into interfering plasmid resulted in remarkable increase of MMEJ efficiency via reducing recombination between two “Repeat” sequences to avoid spacer loss in the mini-CRISPR cassettes (Fig. 2).

### Characteristics of the MMEJ-mediated deletions in *Z. mobilis*

**Fig. 3:**
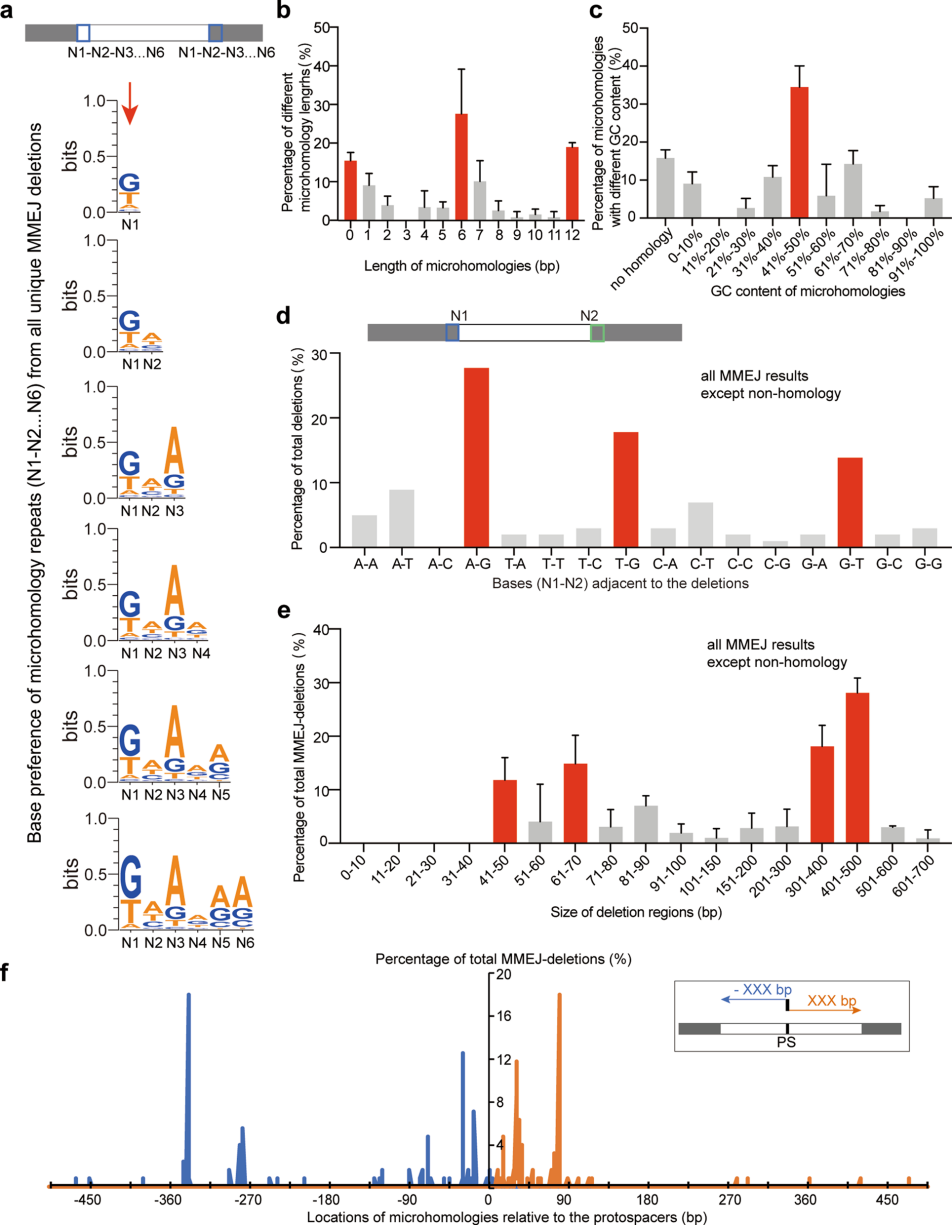
Statistical analysis of the MMEJ deletions in the transformants carrying eight self-interference plasmids. **a** Weblogo analysis of the base preference of the microhomology repeats from all unique MMEJ-mediated deletions. The orders of the bases in the microhomology repeats are indicated. The length of the microhomologies used in each weblogo analysis was ≥ *n* (*n* = 1, 2, 3, 4, 5 or 6). **b** Distribution of microhomology lengths (bp) from all unique MMEJ-mediated deletions. The microhomology lengths is preferred to 0, 6 or 12 bp, marked in red. **c** GC content of the microhomologous sequences from all unique MMEJ-mediated deletions, and it is preferred to 41–50%, marked in red. **d** Conservation of junction bases at the adjacent ends of all unique MMEJ-mediated deletions with the microhomologous repeats (*n* ≥ 1). The 5′- and 3′-adjacnet ends of the deletion region are boxed. The ends are preferred to 5′-A/G-3′, 5′-T/G-3′ and 5′-G/T-3′, marked in red. **e** The deletion sizes of all unique MMEJ-mediated deletions with the microhomologous repeats (N ≥ 1). The sizes are preferred to 41–50 bp, 61–70 bp, 301–500 bp, marked in red. **f** Distances between the microhomology repeats (*N* ≥ 1) and the protospacers. The 5′ upstream (minus) and 3′ downstream (plus) locations of the deletion ends are relative to the 5′-end and 3′ end of the protospacer, respectively

We analysed 119 unique MMEJ-mediated deletions in the transformants carrying the 8 self-interference plasmids (Additional file 6: Table S2). Weblogo analysis of the base preference of the microhomologous repeats showed a relatively conserved 5′-(+ 1)GNANAA(+ 6)-3′ sequence (N = A, T, G or C) in the microhomologous repeat sequences (Fig. 3a). Statistics of N1 included all first bases of the microhomologies with a length of 1–12 bp (101 total sequences), and statistics of N1N2 included all first and second bases of the microhomologies with a length of 2–12 bp (90 total sequences), etc. This result infers that MMEJ system prefer G, A, A and A at + 1, + 3, + 5 and + 6 sites on the microhomology sequences. Moreover, we found ~ 15.1% of all unique deletions (119 total sequences) had no adjacent microhomologous repeats and the longest repeat was 12 bp (Fig. 3b). We also found the deletions in *Z. mobilis* preferred the repeats of 0, 6 or 12 bp in length (Fig. 3b). The microhomology sequences of all unique MMEJ-mediated deletions (119 total sequences) showed no GC content preference (Fig. 3c). We also analysed the base preference at the ends adjacent to the MMEJ-mediated deletions. The result showed that the ends preferred 5′-A/G-3′, 5′-T/G-3′ and 5′-G/T-3′ at the junctions, where the length of the microhomologous repeats ≥ 1 bp (101 total sequences) (Fig. 3d).

Different sizes of deletions were identified in *Z. mobilis* after endogenous CRISPR interference. Here, we sampled all the unique MMEJ-mediated deletions at eight different genes in three independent experiments. The deletions ranged from 4 to 623 bp for all target genes (Additional file 6: Table S2). However, half of all unique deletions (101 total sequences) were 41–70 and 301–500 bp in length (Fig. 3e). Locations of the junction bases joining the deleted regions revealed deletions were bidirectional relative to genomic target sites while showing a evident bias toward the 5′ upstream of the protospacers. We found that 92.1% of the downstream joining bases were located < 100 bp relative to the 3′-end of the protospacers (Fig. 3f). However, 42.5% of the upstream joining bases were found to located far from (> 270 bp) of the 5′-end of the protospacers, and most of the others were located 0–90 bp relative to the 5′-end of the protospacers (Fig. 3f). This result infers that the DNA breaks are processed much stronger toward the 5′ upstream of the protospacers before MMEJ repair.

Because ~ 15.1% of all unique deletions had no adjacent microhomologous repeats (Fig. 3b), we wonder how the end-joining was conducted in these transformants. We analysed all 18 deletions without microhomologies and found that 13 of 18 end joinings exhibited possible microhomologies (2–4 bp) with one or two mutations on the microhomologous repeats, while others (5 of 18 end joinings) showed no possible microhomologies (Additional file 2: Figure S2a). This result suggested most of the end joinings without microhomologies were derived from that with microhomologies but carried mutations probably after the DNA repair process. We also analysed the probability of an end joinings occurred at the same site with the same microhomologies from all identified end joining events. We found ~ 63.4% of end joinings with the microhomologies (*N* ≥ 1) occurred twice or more, while only 11.1% end joinings with no microhomologies occurred twice (Additional file 2: Figure S2b). This result inferred that mutations at the microhomologies occurred randomly. We also analysed the base preference at the ends adjacent to the deletions without microhomologies and found that the ends preferred 5′-A/A-3′, 5′-A/T-3′, 5′-T/A-3′ at the junctions (Additional file 2: Figure S2c). This end base preference differed from that from the deletions with microhomologous repeats (Fig. 3d). The deletions without microhomologies were short in size. The largest sizes were 300–400 bp, and most were shorter than 200 bp (Additional file 2: Figure S2d). Short deletions (0–40 bp) were identified in the deletions without microhomologies, showing much difference compared with the deletions with microhomologies (Additional file 2: Figure S2d and Additional file 3: Figure S3e). Moreover, we found most of the joining bases for the deletions without microhomologies were located 0–90 bp and 0–135 bp relative to the 5′-end and 3′-end of the protospacers, respectively (Additional file 2: Figure S2e), showing no preference toward the 5′ or 3′ direction.

### Characterization of in vivo gene function via CRISPR interference and MMEJ repair

**Fig. 4:**
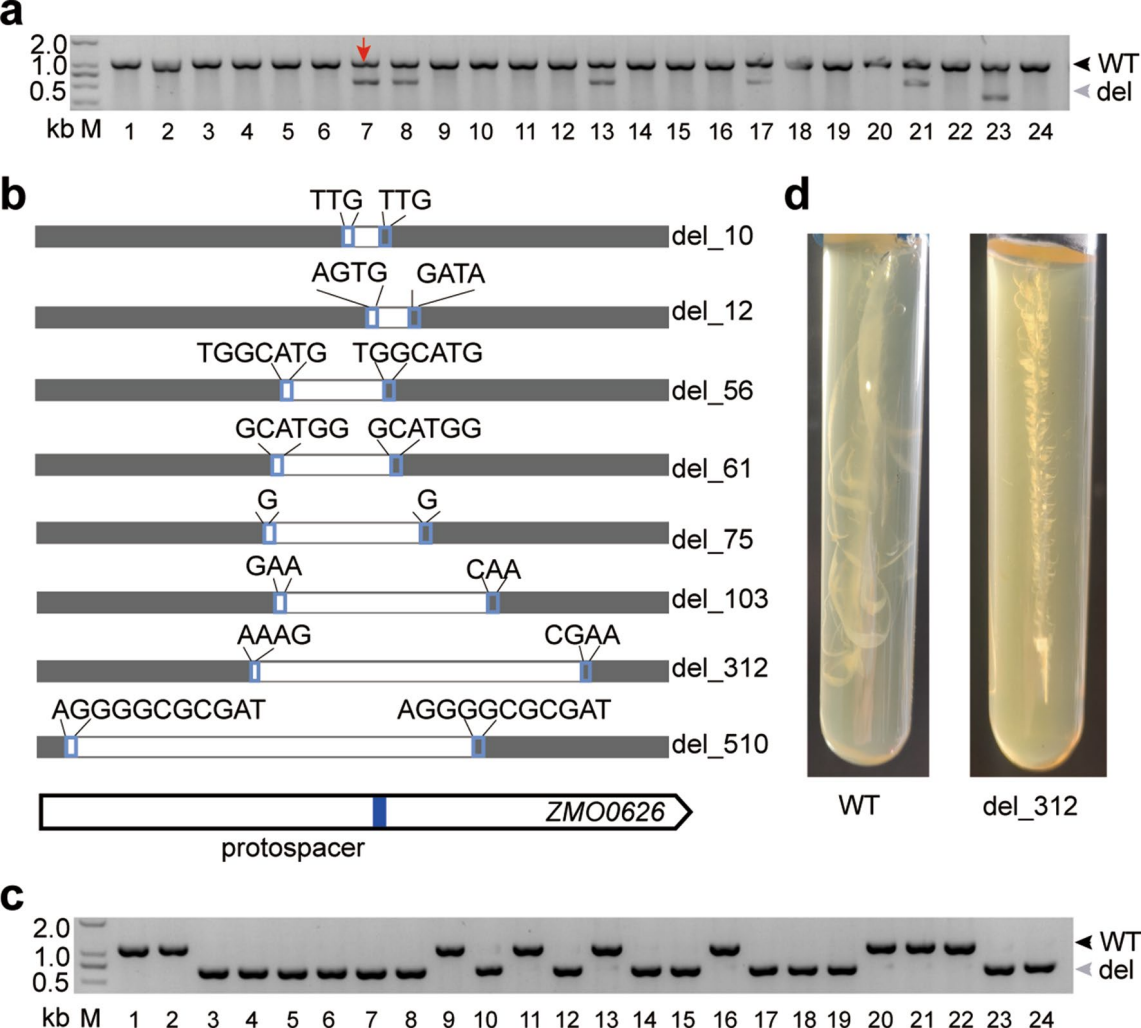
Isolation of a sigma factor encoding gene deletion mutant via CRISPR interference and MMEJ repair. **a** Colony PCR screening for MMEJ mutants transformed with the self-interference plasmid against the *ZMO0626* using primers located upstream or downstream of the target site. Predicted sizes of PCR products from the wild type (wt) and the MMEJ-mediated deletion mutants (del) are indicated, respectively. M, DNA size marker. **b** MMEJ-mediated deletions with different microhomology repeats adjacent to the deletion regions at the σ^28^ factor encoding gene *ZMO0626*. A blue bar on the gene indicates the protospacer location. Grey bars indicate the sequenced regions, white bars and the numbers at the right indicate deletion regions and deletion length (bp). **c** Colony PCR screening for the homozygous deletion mutant colonies isolated by a one-round spreading of the transformant corresponded to lane 1 indicated by a red arrow in **a**. **d** Agar streak test of the mobility of the wild type and the *ZMO0626* mutant. The *ZMO0626* gene encodes a sigma factor (σ^28^) that regulates the transcription of flagella-related genes

Because most of MMEJ-mediated deletions were less than 500 bp in size (Fig. 3f), Agarose gel analysis of the PCR products covering the *ZMO0626* target site, as an example, showed large size and small size bands corresponded to the wildtype and deletion cells, respectively (Fig. 4a). This result indicated that the transformants carrying the interference plasmids were always the mixture of wildtype cells and the MMEJ-mediated deletion cells. We extracted and sequenced the PCR products relative to the small size bands, and identified MMEJ-mediated deletions on the chromosome (Fig. 4b). Different sizes of the deletions (10–510 bp) and microhomologous repeats (1–11 bp) were identified on the chromosome of the transformants (Fig. 4b). The MMEJ-mediated large deletions most probably will lead to loss-of-function (e.g., the transformants del_103, del_312 and del_510), and some short deletions will lead to codon shifts (e.g., the transformants del_10, del_56 and del_51) (Fig. 4b). This result infers that MMEJ-mediated deletion most probably will hinder the gene function in the mutant strain.

The *ZMO0626* gene encodes the σ^28^ factor, which was reported to regulate the transcription of flagella-related genes in *Pseudomonas aeruginosa* [27]. Here, we spread the transformant relative to lane 7 (Fig. 4a) on the agar plate of the RM medium with spectinomycin for isolation of pure deletion colonies. PCR analysis of the colonies of the first passage showed that 15 of 24 colonies carried pure MMEJ-mediated deletions on their chromosomes (Fig. 4c), inferring that a single-round of spread of the transformant efficiently isolated the deletion mutants. Then, we studied the mobility of the MMEJ-mediated *ZMO0626* deletion strain (del_312) in comparison with the wild-type cells in a single-tube agar stab test (Fig. 4d). The wildtype strain showed strong mobility, reflected by the formation of cloud-like structure around the puncture line, while *ZMO0626* gene partial deletion strain showed no mobility in this test (Fig. 4d). This result infers that *ZMO0626* encodes the sigma factor σ^28^ for regulation of mobility-related genes in *Z. mobilis*. It also demonstrates that the gene deletion tool through CRISPR interference and MMEJ repair is feasible for study of the gene functions in vivo.

### Large fragment deletion via expression of two spacers in tandem and subsequent MMEJ repair

**Fig. 5:**
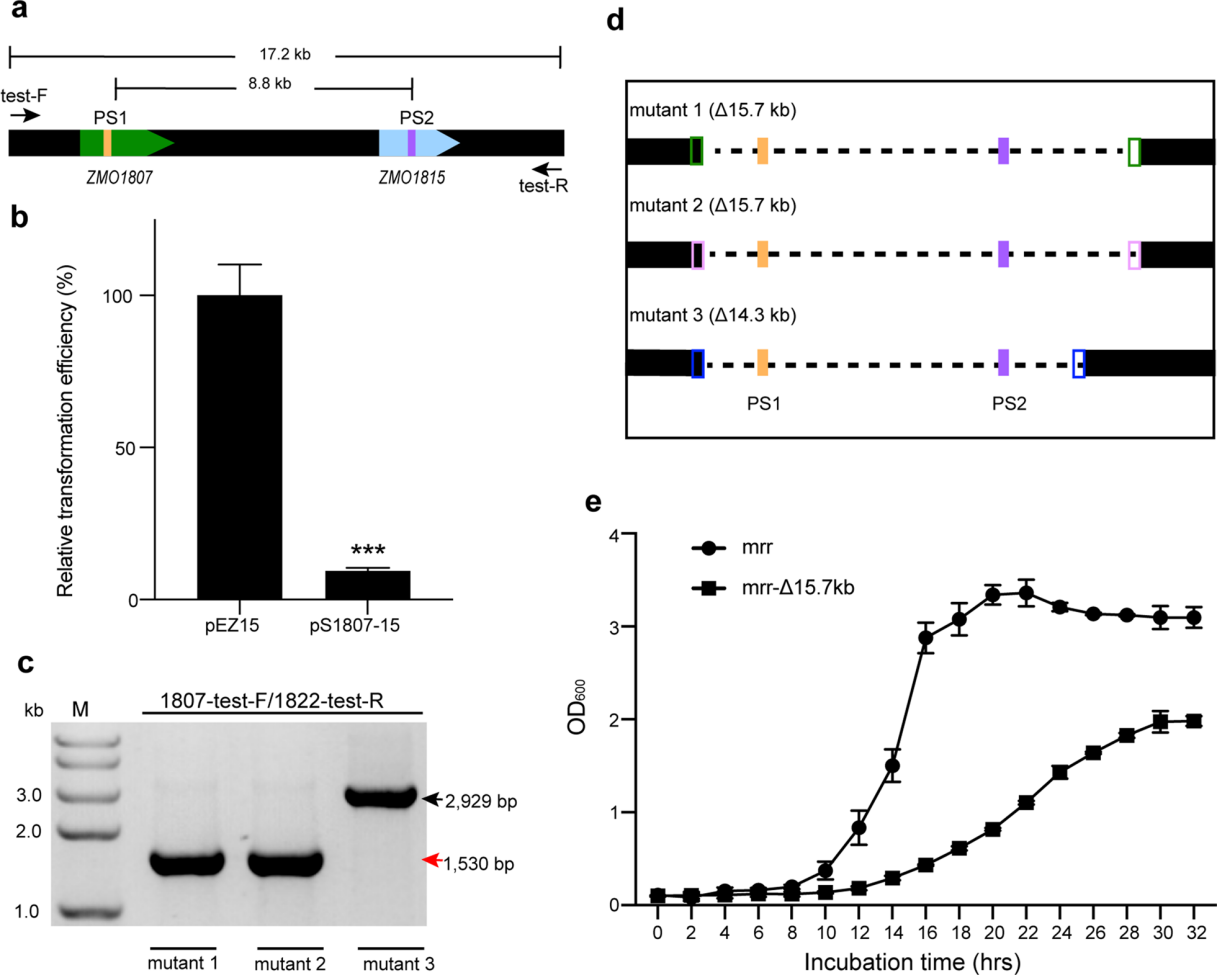
Large fragment deletion via endogenous CRISPR interference and MMEJ repair*.* **a** Schematic of simultaneous targeting by the self-targeting plasmid pS1807-15 carrying “Repeat-Spacer(1807)-Repeat-Spacer(1815)-Repeat” expression cassette against the protospacers at *ZMO1807* and *ZMO1815* gene loci. The PAM sequences for the protospacers and the primers used to amplify the region between two protospacers are indicated. The distance between two protospacers and the primers are shown, respectively. **b** Transformation efficiency of pS1807-15 relative to the empty plasmid pEZ15Asp. Error bars represent the SD of three independent experiments. The significance was determined using a *t* test; *p* < 0.001 ***. **c** Agarose gel analysis of the PCR products covering the regions between two protospacers using the primers 1807-test-F and 1822-test-R from three randomly selected single colonies. The sizes of the PCR products are indicated. M, DNA size marker. **d** Schematic of the MMEJ-mediated deletion regions. The distances between the microhomology repeats to the protospacers and the deletion sizes are indicated, respectively. **e** Growth curves of *Z. mobilis* ZM4mrr strain and the large size deletion strain 1 in the RM medium

We have successfully deleted several individual genes via endogenous CRISPR interference and MMEJ repair. We wondered whether this method could be used to delete large fragment on the genomic DNA. Therefore, we constructed a self-targeting plasmid, pS1807-15 carrying a two spacer-expression cassette in tandem. These spacers target *ZMO1807* and *ZMO1815*, respectively, and the distance between these genes are 8.8 kb on the genomic DNA (Fig. 5a). Transformation efficiency of pS1807-15 plasmid decreased significantly compared with the control empty vector pEZ15Asp (*p* < 0.001; Fig. 5b). Then, we randomly selected 24 single colonies on the plate to verify MMEJ-mediated deletions by PCR using the primers located upstream or downstream of the target genes (Fig. 5a). Two PCR products of ~ 1.5 kb and one of ~ 2.9 kb was obtained (Fig. 5c). These PCR products were much shorter than expected on the wild-type genomic DNA (17.2 kb), indicating that large DNA fragments were deleted via this method on the genome of the transformants. Sequencing of these PCR products indeed confirmed the 14.3–15.7 kb deletions at different joining sites in these transformants (Fig. 5d). PCR verification also demonstrated that the efficiency of MMEJ-mediated large fragment deletion was 8.3% in three independent transformation experiments. The low transformation efficiency and editing efficiency may be relate to the importance of the deleted genes. For example, the deleted gene *ZMO1816* encodes a Fis family transcriptional regulator, which is thought to plays a wide range of roles, including regulation of chromosome replication, DNA transcription and recombination [28]. The growth rate and maximum bacterial density of the mutant strain were significantly lower than that of the wildtype cells (Fig. 5e), further indicating the importance of the deleted genes.

## Discussion

Traditional gene deletion method requires homologous donor DNAs for homologous recombination to remove the target gene from the genome [19]. Although sequence-specific endonuclease-based method could introduce indel mutations at the target gene locus through non-homologous end joining (NHEJ) in eukaryotic cells [29], accurate target gene deletion also requires homologous donors. Furthermore, NHEJ pathway is present in a small number of bacterial genomes [30, 31], making it less applicable in prokaryotes. *Zymomonas mobilis* is a model system for production of biofuels and biochemicals [1, 2]. Recently, an endogenous CRISPR- and homologous directed recombination (HDR)-based genome editing tool has been developed for *Z. mobilis* [19]. This system requires an editing plasmid carrying an endogenous derived “Repeat-Spacer-Repeat” expression cassette and the homologous upstream/downstream donors. Although this tool is efficient for genome editing, large scale or high-throughput in vivo gene function study requires simpler genetic method. Most recently, we have discovered MMEJ pathway is employed to repair the endogenous CRISPR-cleaved genomic DNA in *Z. mobilis* [25]. MMEJ repair leads to deletions at the target gene locus with different sizes. Based on MMEJ pathway, we developed a simpler editing tool only requiring an endogenous derived “Repeat-Spacer-Repeat” expression cassette in the editing plasmid. Transformation of this editing plasmid results in desired deletions at the target loci in the transformants (Figs. 1c and 4b). The deletion size mainly ranges from 40 to 500 bp (Fig. 3e), that is enough to hinder the gene function. Given the difference in MMEJ efficiency caused by targeting different genes, we proposed that targeting sites adjacent to putative essential genes would also affect its editing efficiency. For example, MMEJ repair efficiency at the target in *ZMO1822* gene was remarkable lower than that of other genes (Fig. 3c and Additional file 4: Figure S4). The neighbor genes, including *ZMO1823* and *ZMO1824*, encode the proteins involved in electron transport, and were probably the essential genes [32]. The importance of these genes might account for the low MMEJ repair efficiency found in the single colonies after transformation of the self-targeting plasmid against *ZMO1822* gene. Recently, MMEJ-mediated deletions were also identified in *Pseudomonas aeruginosa*, *Escherichia coli*, *Pseudomonas syringae* and *Klebsiella pneumoniae* post subtype I–C CRISPR interference [33]. In striking contrast to our results, most of the MMEJ-mediated deletions in these species were larger than 5 kb [33], revealing that the subtype I–C and MMEJ-mediated genetic tool is feasible for genome reduction but hard for single specific gene editing. In our study, individual genes could be edited via MMEJ to verify their functions. For example, MMEJ-mediated partial deletion of σ^28^-conding gene resulted in loss of mobility (Fig. 4d), conforming the regulatory function of σ^28^ in *Z. mobilis*.

To harness MMEJ tool more efficiently and conveniently, we identified two key factors that affect CRISPR and MMEJ efficiencies. First, we found that CRISPR targeting at the protospacer with lower GC content showed higher MMEJ-mediated deletion efficiency and vice versa (Fig. 1d). We propose that high GC content probably affected the DNA end resection at break by repair nucleases [21]. Therefore, we suggest to select the protospacers with lower GC content (< 50%) when using the MMEJ pathway for gene deletion in *Z. mobilis* and probably other species showing MMEJ activity [33]. Moreover, spacer excision at the gRNA expression cassette was identified in different CRISPR-Cas systems [34] (Fig. 2b), probably due to recombination between the two repeats in the “Repeat-Spacer-Repeat” expression cassette. Therefore, we constructed an editing plasmid with engineered second repeat of the mini-CRISPR cassette to reduced spacer excision and to increase MMEJ-mediated deletion efficiencies (Fig. 2f).

Because CRISPR interference and subsequent MMEJ repair result in short deletions in target gene loci (Fig. 3e), making it possible to screen the essential genes and study their functions in vivo. For example, the *ligase-A* (*ligA*, *ZMO0364*) gene, which encodes a DNA ligase involved in the ligation of the Okazaki fragment [21], could not be deleted in this study via CRISPR- and HDR-based gene deletion method [19]. CRISPR targeting at different protospacers in the coding region of *ligA* gene resulted in very low transformation efficiencies and resulted in no MMEJ-mediated deletions on the genomes (Additional file 3: Figure S3a), strongly inferring the essentiality of *ligA* gene. Moreover, targeting at the *ligA* promoter region resulted in significantly lower transformation efficiency compared with the control empty vector pEZ15Asp (*p* < 0.001) and identification of an 84-bp deletion in the promoter region (Additional file 3: Figure S3a, b). This transformant grew significantly slower than the wildtype strain (Additional file 3: Figure S3c), confirming the importance of *ligA* gene in *Z. mobilis*. Our result infers that, compared to regular gene deletion approaches [17, 19], the endogenous CRISPR- and MMEJ-mediated tool is feasible to screen essential genes at a large scale in *Z. mobilis*. Moreover, MMEJ-mediated deletion at the promoter region of the essential gene could affect its transcription and function (Additional file 3: Figure S3c), similar to the CRISPRi method [35] but requiring less construction of editing plasmids and resulting in higher genetic stable strain for further study.

## Conclusions

The tool we have established in this study enables rapid and efficient genome editing in *Z. mobilis*, including deletion of single genes, deletion of large-scale DNA fragments and the verification of essential genes. This tool only requires a self-interference plasmid for expressing the guide RNA. Thus, we provide a powerful toolkit for diverse genome engineering purposes, which greatly benefit further study in this species*.* Moreover, we summarized the characters of MMEJ-mediated deletions, and points out the distinct mechanisms of MMEJ pathway in *Z. mobilis* and other species.

## Materials and methods

### Strains and growth conditions

*Zymomonas mobilis* strains, including ZM4mrr lacking an endonuclease gene *ZMO0028* [36], ZM4mrr carrying plasmids and ZM4mrr derived mutants were cultured at 30 °C in RM medium (20 g/L glucose, 10 g/L yeast extract, and 2 g/L K_2_HPO_4_). The *E. coli* strain Trans10 used as the host strain for molecular cloning and manipulation of plasmids was cultured at 37 °C in Luria–Bertani medium. Spectinomycin was added to the *Z. mobilis* and trans10 culture to a final concentration of 100 μg/mL if required.

### Plasmid construction

Construction of the interference plasmids were carried out as described previously [25]. In general, the self-targeting plasmids (pS) carrying the expression cassette of “Repeat-Spacer-Repeat” against the genes (*ZMO0626, ZMO0631, ZMO0672, ZMO1063, ZMO1404, ZMO1807, ZMO1815, ZMO1822* genes) were constructed by cloning the 32-bp sequences following NCC (*N* = any nucleotide) PAMs from these genes coding sequences into the pEZ15Asp vector [8] under control of the *cas1* gene promoter (P*cas1*), resulting in interference plasmids pS0626, pS0631, pS0672, pS1404, pS1063, pS1807, pS1815, pS1822. Modified self-targeting plasmids were constructed by introducing mutations into plasmid pS using whole-plasmid amplification with mutated primers and TEDA technology [37]. All plasmids were transformed into *E. coli* Trans 10 and extracted using the Plasmid Mini Kit I (Omega). All primers used are listed in Additional file 5: Table S1.

### Gene deletion and mutant identification

The self-targeting plasmids were electroporated into *Z. mobilis* ZM4mrr, and the transformants were selected on RM medium agar plates with 100 μg/mL spectinomycin. PCR was used to amplified the regions covering the targeting sites on the chromosome using the primers upstream or downstream apart from the target sites (Additional file 5: Table S1). The PCR products were visualized on 1.5% agarose gels and were sequenced to identify the deletions covering the target sites.

### Measurement of growth curves

A single colony from the streaked plate was transferred to the RM medium, and cultivated to OD_600_ = 1.0 as seed solution. The seed solution (1%) was transferred to 100 mL RM medium, and incubated at 30°C. Samples are taken every 2 h for test of the OD_600_ values until the culture reached stationary state.

### Agar streak test

Agar streak tests were performed on semi-solid agar tubes, as described previously [38], to evaluate the motility of *Z. mobilis* strains. Bacterial isolates were inoculated by vertical stab culture using inoculation needles into 25 mL tubes containing 15 mL semi-solid RM medium, which solidified with 0.5% agar. After that, the tubes were incubated at 30°C for 3 days.

## Supplementary Information

**Additional file 1: Figure S1.** Mutation at the loop and non-structured sequence on the second repeat of the mini-CRISPR reduced spacer deletion.

**Additional file 2: Figure S2.** Statistical analysis of the deletions without microhomologies.

**Additional file 3: Figure S3.** Identification of essential gene *ZMO0364* (*ligase-A*).

**Additional file 4: Figure S4.** High GC content of the protospacers reduced the efficiency of MMEJ-mediated deletion in *Z. mobilis*.

**Additional file 5: Table S1.** Primers used in this study.

**Additional file 6: Table S2.** MMEJ-mediated deletions and corresponding micro homologous repeats.

## Data Availability

All data generated or analysed during this study are included in this published article and its supplementary information files.

## Abbreviations

CRISPR: Clustered regularly interspaced short palindromic repeats
MMEJ: Microhomology-mediated end joining
HDR: Homology-directed repair
NHEJ: Non-homologous end joining
ED: Entner–Doudoroff
RM: Rich medium
