# Supplementary material for: Endogenous CRISPR-assisted microhomology-mediated end joining enables rapid genome editing in Zymomonas mobilis
Source: Biotechnol Biofuels. 2021 Oct 24;14:208. doi: 10.1186/s13068-021-02056-z (PMC8543907; doi:10.1186/s13068-021-02056-z)
Supplement: Supplementary file 1 — Additional file 1: Figure S1. Mutation at the loop and non-structured sequence on the second repeat of the mini-CRISPR reduced spacer deletion. [file 13068_2021_2056_MOESM1_ESM.pdf]

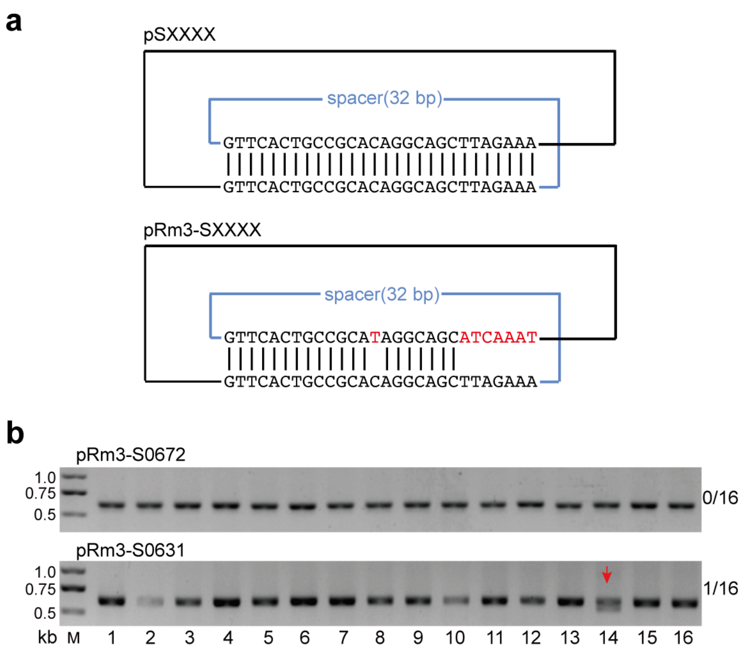

Supplementary Figure 1. Mutation at the loop and non-structured sequence on the second repeat of the mini-CRISPR reduced spacer deletion. (a) Schematic of spacer-excision result. Sequencing result of spacer loss from the self-targeting plasmid and the complete artificial-CRISPR array. Engineered modified nucleotides and mutated nucleotides shown in red; repeat sequences highlighted in grey with an intervening spacer sequence. **(b)** Detection of spacer excision at artificial mini-CRISPR of pRm-S0672(above) and pRm-S0631(below) on 1.5% agarose gel. Each colony of transformant was cultured in liquid medium for 1 day and amplified using primer set of pS-test-F/pS-test-R. Small bands of electrophoresis indicated incomplete mini-CRISPR for excision of spacer, marked by red arrows. These data represent no spacer-excision transformant observed in pRm3-S0672(above) and one spacer-excision transformant observed in pRm3-S0631(below).
