# Supplementary material for: Endogenous CRISPR-assisted microhomology-mediated end joining enables rapid genome editing in Zymomonas mobilis
Source: Biotechnol Biofuels. 2021 Oct 24;14:208. doi: 10.1186/s13068-021-02056-z (PMC8543907; doi:10.1186/s13068-021-02056-z)
Supplement: Supplementary file 2 — Additional file 2: Figure S2. Statistical analysis of the deletions without microhomologies. [file 13068_2021_2056_MOESM2_ESM.pdf]

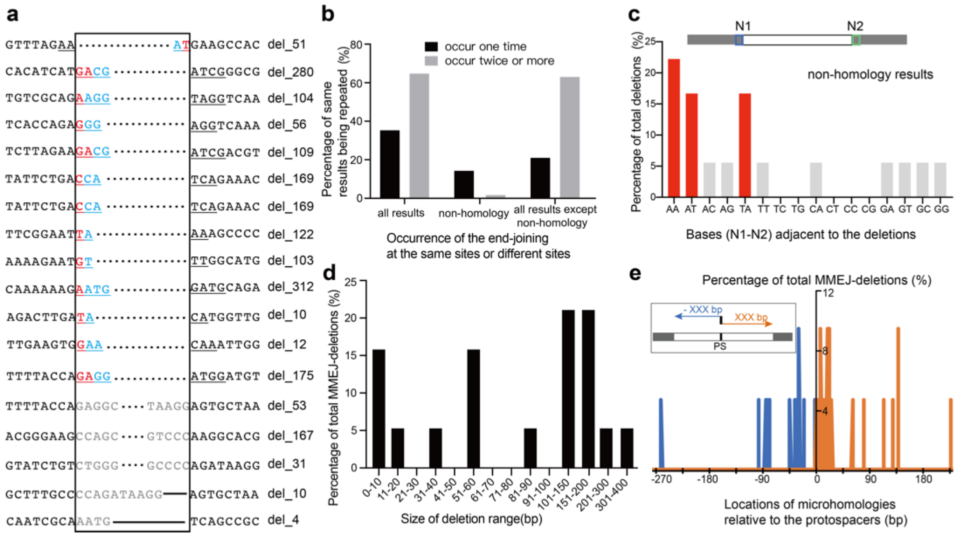

Supplementary Figure 2. Statistical analysis of the deletions without microhomologies. (a) Diagram of the total 18 sequencing results of 0 bp-homology-mediated end joining. The possible microhomologies are underlined, and the possible mutant sites are marked in red. Deletion regions are boxed out, and their sizes are listed on the right. Dash and solid lines in the sequences: omitted bases and no omitted bases, respectively. **(b)** The occurrence of the end joining at the same sites or different sites. **(c)** The conservation of first bases at the end of 0 bp-homology-mediated deletions. The 5'-end and 3'-end of the deletion regions are boxed. **(d)** The distribution of diverse deletion sizes (bp) of all 0 bp-homology-mediated end joining. **(e)** The distances between the deletion ends and the protospacers. The 5' upstream (minus) and 3' downstream (plus) locations of the deletion ends are relative to the 5'-end and 3' end of the protospacer, respectively.
