# Supplementary material for: Endogenous CRISPR-assisted microhomology-mediated end joining enables rapid genome editing in Zymomonas mobilis
Source: Biotechnol Biofuels. 2021 Oct 24;14:208. doi: 10.1186/s13068-021-02056-z (PMC8543907; doi:10.1186/s13068-021-02056-z)
Supplement: Supplementary file 3 — Additional file 3: Figure S3. Identification of essential gene ZMO0364 (ligase-A). [file 13068_2021_2056_MOESM3_ESM.pdf]

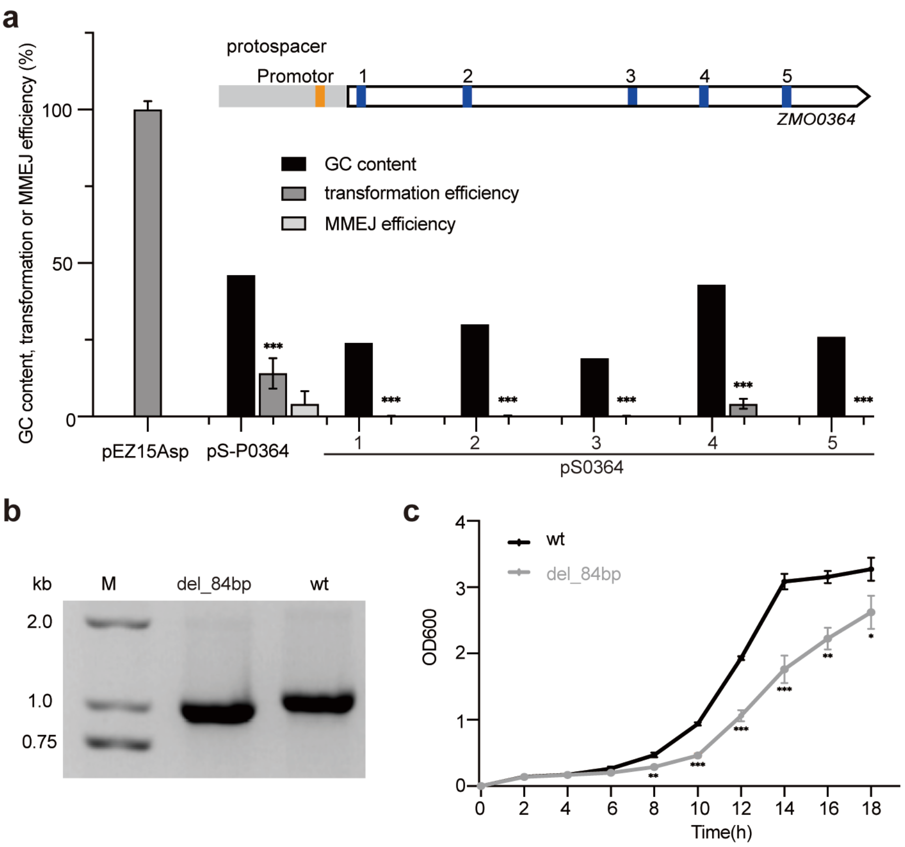

Supplementary Figure 3. Identification of essential gene *ZMO0364* (*ligase-A*). (a) *ZMO0364* could not be edited at any region, whereas its *promotor* region could be edited. five protospacers of *ZMO0364* and one protospacer of *promotor* region are shown in the above, and their corresponding GC content, transformation efficiency, MMEJ efficiency are shown in the below. Error bars represent the SD of three independent experiments. The significance was determined using a *t*-test; *p*<0.05 *, *p*<0.01 **, *p*<0.001 ***. **(b)** Colony PCR screening for MMEJ mutant transformed with the pS-P0364 against the *promotor* region of *ZMO0364*. The size of PCR product from wide-type (wt) is also shown in the agarose gel electrophoresis. **(c)** Growth curves based on absorbance at 600 nm of wide-type (wt) and del_84bp. Error bars represent the SD of three independent experiments. The significance was determined using a *t*-test; *p*<0.05 *, *p*<0.01 **, *p*<0.001 ***.
