# Supplementary material for: Endogenous CRISPR-assisted microhomology-mediated end joining enables rapid genome editing in Zymomonas mobilis
Source: Biotechnol Biofuels. 2021 Oct 24;14:208. doi: 10.1186/s13068-021-02056-z (PMC8543907; doi:10.1186/s13068-021-02056-z)
Supplement: Supplementary file 4 — Additional file 4: Figure S4. High GC content of the protospacers reduced the efficiency of MMEJ-mediated deletion in Z. mobilis. [file 13068_2021_2056_MOESM4_ESM.pdf]

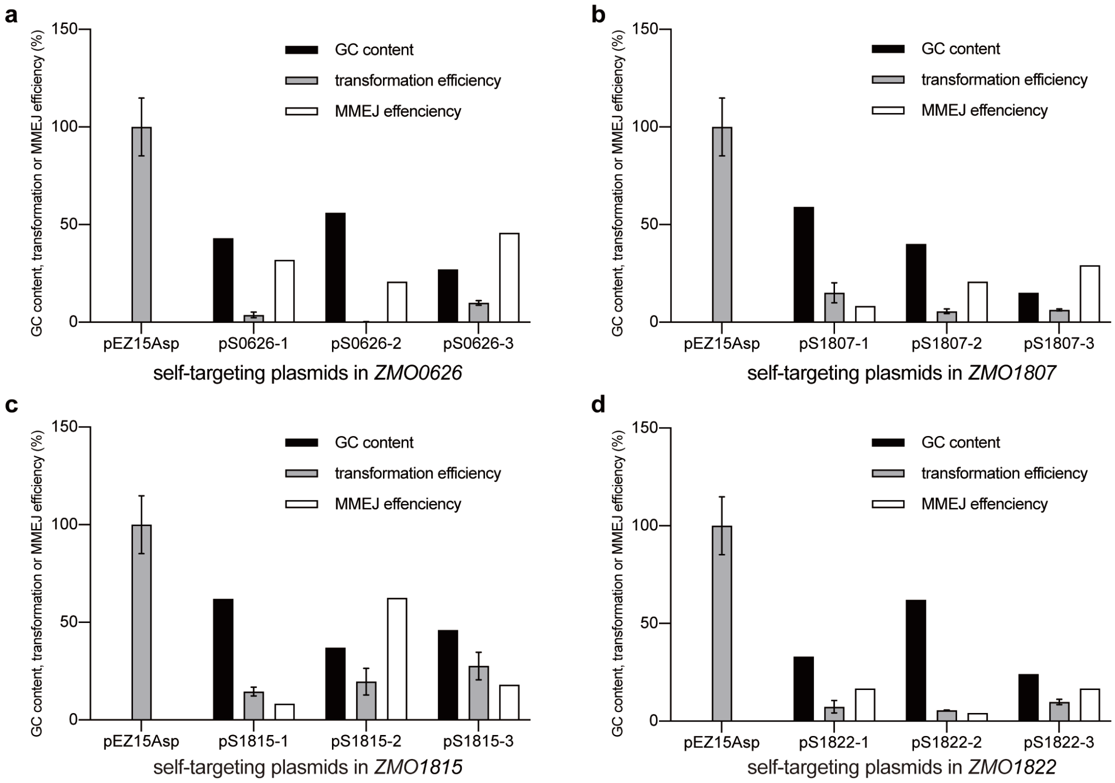


**Supplementary Figure 4. High GC content of the protospacers reduced the efficiency of MMEJ-mediated deletion in *Z. mobilis*.** Three protospacers with different GC content on *ZMO0626*(**a**), *ZMO1807*(**b**), *ZMO1815*(**c**), *ZMO1822*(**d**) genes were selected as the targets, respectively. GC content, transformation efficiency, MMEJ efficiency were shown in black, grey and white.
