# Supplementary material for: Endogenous CRISPR-assisted microhomology-mediated end joining enables rapid genome editing in Zymomonas mobilis
Source: Biotechnol Biofuels. 2021 Oct 24;14:208. doi: 10.1186/s13068-021-02056-z (PMC8543907; doi:10.1186/s13068-021-02056-z)
Supplement: Supplementary file 5 — Additional file 5: Table S1. Primers used in this study. [file 13068_2021_2056_MOESM5_ESM.pdf]

Supplementary Table 1: primers used in this study.

Primers	Sequences (5′ to 3′)
**Primer for cloning**	
pS(TEDA)-F	GCGGCCGCTTCTAGAATTTAC
pS(TEDA)-R	GGGTAAATTCAATTTGCGCCATTATTCTCGTCCTTAAAACAGAGGCC
pRmS1(TEDA)-F	TTTCTAAGCTCGGTGTTCCCGAGTGAACTGAGACCGACG
pRmS1(TEDA)-R	AACACCGAGCTTAGAAAGGATCCTCGAAC
pRmS2(TEDA)-F	TAAGGACGGTTTTCCCGTCTGAACTGAGACCGACGTC
pRmS2(TEDA)-R	GGAAAACCGTCCTTAGAAAGGATCCGGATCCTC
pRmS3(TEDA)-F	TAGGCAGCATCAAATGGATCCTCGAACGCGCCGAATA
pRmS3(TEDA)-R	CATTTGATGCTGCCTATGCGGCAGTGAACTGAG
pRmS4(TEDA)-F	CATAGGGTGCATCAAATGGATCCTCGAACGCGCCGAA
pRmS4(TEDA)-R	TTGATGCACCCTATGCGGGTGTGAACTGAGACCGACGT
pRmS5(TEDA)-F	TCTGGTACCGCAGCTTAGAAAAGAGACCG
pRmS5(TEDA)-R	AAGCTGCGGTACCAGAAAATCTTCTGTATCTACAATG
**Primer for host DNA interference**	
0626Spacer-F	GAAAGTGTTTCTTCTGCGATTGAAGTGGAAGACTTG
0626Spacer-R	GAACCAAGTCTTCCACTTCAATCGCAGAAGAAACAC
0631Spacer-F	GAAAAAGCTGTTGCGTGTTCTCCAAGAACAAGAAGT
0631Spacer-R	GAACACTTCTTGTTCTTGGAGAACACGCAACAGCTT
0672Spacer-F	GAAATAGTGATAAAAATGTCTTAGAAGACGCTTTGT
0672Spacer-R	GAACACAAAGCGTCTTCTAAGACATTTTTATCACTA
1063Spacer-F	GAAAGTTTACAAAAGATCGAAGCTAACTGGTTGGAT
1063Spacer-R	GAACATCCAACCAGTTAGCTTCGATCTTTTGTAAAC
1404Spacer-F	GAAAAACGGCACAATCGCAAATGTCAGCCGCTTCTT
1404Spacer-R	GAACAAGAAGCGGCTGACATTTGCGATTGTGCCGTT
1807Spacer-F	GAAAATTGCCGGAAAAATGGTTCTTGATTTGCAGCA
1807Spacer-R	GAACTGCTGCAAATCAAGAACCATTTTTCCGGCAAT
1815Spacer-F	GAAAAGCATAGCTTTGCCCCAGATAAGGAGTGCTAA
1815Spacer-R	GAACTTAGCACTCCTTATCTGGGGCAAAGCTATGCT
1822Spacer-F	GAAAACGAAATATAATAGGATTGGTGGTTATCCGGC
1822Spacer-R	GAACGCCGGATAACCACCAATCCTATTATATTTCGT
0672Spacer1-F	GAAAGATCATCCAGACCTTGAAAAAGGCGTTGCGGC
0672Spacer1-R	GAACGCCGCAACGCCTTTTTCAAGGTCTGGATGATC
0672Spacer2-F	GAAATTAACTCTGAATAGCTTGCAAAAGCTATTCTT
0672Spacer2-R	GAACAAGAATAGCTTTTGCAAGCTATTCAGAGTTAA
0672Spacer4-F	GAAATGAAGGTTTTCGTCGTAATAGCTATCGTAAAT
0672Spacer4-R	GAACATTTACGATAGCTATTACGACGAAAACCTTCA
0672Spacer5-F	GAAAGGGTATTGGCCCAAGTCGGAAGAAGGCTTTGC

0672Spacer5-R	GAACGCAAAGCCTTCTTCCGACTTGGGCCAATACCC
0364(P)Spacer-F	GAAATGAAAAACAGGATTTGTCAGCTCTGGCTCCGA
0364(P)Spacer-R	GAACTCGGAGCCAGAGCTGACAAATCCTGTTTTTCA
0364Spacer1-F	GAAAATTACGATCATCTTTATCATGATAAGGATAAT
0364Spacer1-R	GAACATTATCCTTATCATGATAAAGATGATCGTAAT
0364Spacer2-F	GAAAAAAACATTAAAAGGCGATAATTGGCCAGAAAT
0364Spacer2-R	GAACATTTCTGGCCAATTATCGCCTTTTAATGTTTT
0364Spacer3-F	GAAATGGCGTTGCACCGGAGGTTTGATCTGTCCAGC
0364Spacer3-R	GAACGCTGGACAGATCAAACCTCCGGTGCAACGCCA
0364Spacer4-F	GAAAATTGATGAGGCGGCTCATCTACGGACTATATT
0364Spacer4-R	GAACAATATAGTCCGTAGATGAGCCGCCTCATCAAT
0364Spacer5-F	GAAAGCAGGTTCTAAGCTTTCAAAAGCGAAAGAGCT
0364Spacer5-R	GAACAGCTCTTTCGCTTTTGAAAGCTTAGAACCTGC
0626Spacer2-F	GAAAATCCGCCCATTCGCGATATTCGCTGGGAGACA
0626Spacer2-R	GAACTGTCTCCCAGCGAATATCGCGAATGGGCGGAT
0626Spacer3-F	GAAATTGGTTTTACAGCTTTATTATGTCGAAGAATT
0626Spacer3-R	GAACAATTCTTCGACATAATAAAGCTGTAAAACCAA
1807Spacer1-F	GAAAGAGCGTTTGGCGCCTTCCTCCGTACCTCTGAA
1807Spacer1-R	GAACTTCAGAGGTACGGAGGAAGGCGCCAAACGCTC
1807Spacer3-F	GAAATCAATTACAATGTTTTTATAAATATAATCCAA
1807Spacer3-R	GAACTTGGATTATATTTATAAAAACATTGTAATTGA
1815Spacer1-F	GAAAAGCCGGTGCCGTCATGCCCTGCTGAATACTGC
1815Spacer1-R	GAACGCAGTATTCAGCAGGGCATGACGGCACCGGCT
1815Spacer2-F	GAAATAAAGAAAAACTCTGGCTACTGGTTGGGTTAT
1815Spacer2-R	GAACATAACCCAACCAGTAGCCAGAGTTTTTCTTTA
1822Spacer1-F	GAAAAAAATGCTTGCCAAAACTCTTGGTGATATAGA
1822Spacer1-R	GAACTCTATATCACCAAGAGTTTTGGCAAGCATTTT
1822Spacer2-F	GAAACCGCTCGCCGGACTGGTATTGCCCCGAAACAT
1822Spacer2-R	GAACATGTTTCGGGGCAATACCAGTCCGGCGAGCGG
1822Spacer3-F	GAAATTAATGATAAATTATCAGAAAAATGATGGACG
1822Spacer3-R	GAACCGTCCATCATTTTTCTGATAATTTATCATTAA
1815-test-F	CCGGCCTTATTCCAGCTTG
1815-test-R	GGAACTTCCCGCCCTCAAT
1822-test-F	ATATCGCCCGAATTGCG
1822-test-R	GATTATAAGATGTTTCGGGGC
0672-1-test-F	GATGCCAAGATTCAATCCCAG
0672-1-test-R	TCACTAGGGAGATAATTGGTCAGAA
0672-2-test-F	ACGAATCCCAAGAAACAGATCG
0672-2-test-R	ACAAAGCGTCTTCTAAGACATTTTTATCACTA
0672-4-test-F	CGATTAACAGCGGCAATTTAAGAA
0672-4-test-R	GCCGAGGAAATCCCATTTACTT
0672-5-test-F	GACAGAATCGAGGTGTATGACAA
0672-5-test-R	GCCAATAACCTTTTCAGATTTTGGA

0364-P-test-F	TTTACATGGGATGGGCCTATC
0364-P-test-R	ATAATTTCTGGCCAATTATCGCC
0364-1-test-F	CAAGGCTGGAGCTTAGCAG
0364-1-test-R	TGCCCGATGTGCAATTTTG
0364-2-test-F	AGGCTGTCATTTGCACTGT
0364-2-test-R	CTTGCGGTAATATTCGGGTCTA
0364-3-test-F	GGAGATCGGGTTCTGGTTCA
0364-3-test-R	AAGCTGACGTTTTTGAAACAACC
0364-4-test-F	GGTTGGGGTGAACTTTCTGT
0364-4-test-R	CAGAAACAACATCGGAATTATGGG
0364-5-test-F	GTTGGCCTCTTCACCTTTG
0364-5-test-R	TTGCGTCGATCATCTACGAT
16s-test-F	AGTGTGGCTGATCATCCTC
16s-1-test-R	CCTTTGCGCTGAAGGTCTTTA
16s-2-test-R	CTGTCAGAGGAATAAGTTACGTAAC
16s-3-test-R	TCGAATTTATCGAGGATGCCTC
pS-test-F	GGTTATATACACGGGCAAAGCCA
pS-test-R	ATAAACGCAGAAAGGCCCAC
pRmS1-test-R	TAAGCTCGGTGTTCCCGAG
pRmS2-test-R	TAAGGACGGTTTTCCCGTCT
pRmS3-test-R	TCCATTTGATGCTGCCTA
pRmS4-test-R	CCATTTGATGCACCCTAT
pRmS5-test-R	TTTTCTAAGCTGCGGTACCAGAA
