# Supplementary material for: Endogenous CRISPR-assisted microhomology-mediated end joining enables rapid genome editing in Zymomonas mobilis
Source: Biotechnol Biofuels. 2021 Oct 24;14:208. doi: 10.1186/s13068-021-02056-z (PMC8543907; doi:10.1186/s13068-021-02056-z)
Supplement: Supplementary file 6 — Additional file 6: Table S2. MMEJ-mediated deletions and corresponding micro homologous repeats. [file 13068_2021_2056_MOESM6_ESM.pdf]

Supplementary Table 2: MMEJ-mediated deletions and corresponding micro homologous repeats

No.	Size of deletions(bp)	Sequence of microhomologies	Length of microhomologies(bp)	Target genes	Occurrence times
1	4	none	0	ZMO1404	1
2	10	none	0	ZMO1815	1
3	10	none	0	ZMO0626	1
4	12	none	0	ZMO0626	1
5	31	none	0	ZMO1815	1
6	51	none	0	ZMO1807	1
7	53	none	0	ZMO1815	1
8	56	none	0	ZMO1822	1
9	103	none	0	ZMO0626	1
10	104	none	0	ZMO1822	1
11	109	none	0	ZMO0672	1
12	122	none	0	ZMO1404	1
13	167	none	0	ZMO1404	1
14	169	none	0	ZMO0672	2
15	175	none	0	ZMO1815	1
16	280	none	0	ZMO1815	1
17	312	none	0	ZMO0626	1
18	54	G	1	ZMO1404	1
19	55	G	1	ZMO1807	1
20	75	G	1	ZMO0626	1
21	86	T	1	ZMO1807	6
22	153	A	1	ZMO1404	1
23	529	C	1	ZMO1807	1
24	79	GG	2	ZMO0631	1
25	139	AA	2	ZMO1063	1
26	152	TA	2	ZMO1807	1
27	307	CG	2	ZMO1807	1
28	623	AA	2	ZMO1807	1
29	82	AAC	3	ZMO1807	1
30	50	CGGA	4	ZMO1807	1
31	213	AGGT	4	ZMO1822	1
32	426	AAAA	4	ZMO1807	1
33	48	CGAAG	5	ZMO1063	2
34	74	ATGTC	5	ZMO1404	1
35	90	GCAAT	5	ZMO0672	1
36	487	CCAAC	5	ZMO1822	1
37	47	TTAGCC	6	ZMO1063	9
38	61	GCATGG	6	ZMO0626	15
39	255	AAGAAG	6	ZMO0631	1

40	313	TTGGAA	6	ZMO1815	7
41	381	AAGCCA	6	ZMO0631	1
42	510	ATTCTG	6	ZMO0672	1
43	56	TGGCATG	7	ZMO0626	2
44	212	GCCACAG	7	ZMO1822	1
45	312	GCTGGCA	7	ZMO1815	1
46	321	TATCGAG	7	ZMO0672	5
47	423	GAAAAAG	7	ZMO0672	3
48	191	AAGGAATC	8	ZMO1822	1
49	366	GGCATAGC	8	ZMO1822	2
50	372	CAGCGTTTC	9	ZMO1404	1
51	99	TTAGAAGACG	10	ZMO0672	2
52	510	AGGGGCGCGAT	11	ZMO0626	1
53	423	GAAAAAGCTGGT	12	ZMO0672	23
